# Editorial: Intestinal Dysbiosis in Inflammatory Diseases

**DOI:** 10.3389/fimmu.2021.727485

**Published:** 2021-07-30

**Authors:** Gislane Lelis Vilela de Oliveira, Cristina Ribeiro de Barros Cardoso, Veena Taneja, Alessio Fasano

**Affiliations:** ^1^Microbiology Program, Institute of Biosciences, Humanities and Exact Sciences, São Paulo State University, Sao Jose do Rio Preto, Brazil; ^2^Department of Food Engineering and Technology, Institute of Biosciences, Humanities and Exact Sciences, São Paulo State University, Sao Jose do Rio Preto, Brazil; ^3^Department of Clinical Analysis, Toxicology and Food Sciences, School of Pharmaceutical Sciences of Ribeirão Preto, University of São Paulo, Ribeirão Preto, Brazil; ^4^Department of Immunology and Department of Medicine, Division of Rheumatology, Mayo Clinic Rochester, Rochester, MN, United States; ^5^Division of Pediatric Gastroenterology and Nutrition, Center for Celiac Research, Mucosal Immunology and Biology Research Center, Massachusetts General Hospital, Boston, MA, United States; ^6^Gastroenterology and Nutrition, Harvard Medical School, Boston, MA, United States; ^7^European Biomedical Research Institute of Salerno, Salerno, Italy

**Keywords:** inflammatory diseases, gut microbiota, dysbiosis, gut permeability, microbiota modulators

The human body is densely colonized by more than a trillion of commensal and mutualistic microorganisms, including archaea, bacteria, virus, bacteriophages and fungi (1–3). The majority of these microbial communities, referred to as the microbiota, resides within the intestine and is influenced by several factors that shape the establishment of a predominant protective, diverse or potentially harmful population in this ecosystem. As an example, the mode of delivery exposes the neonate to different microorganisms depending on the birth route. Vaginal-delivered babies are usually colonized by a more protective microbiota, while C-section newborns first contact common skin bacteria. Similarly, breastfeeding is mostly advantageous because of the immune protection mediated by antibodies, lactoferrin and anti-microbial peptides present in human milk, besides the known gut protective microorganisms *Bifidobacterium* and *Lactobacillus* that usually are not present in infant formulas. These factors may protect the newborn to infections, allergies and other future morbidities. In addition, by the introduction of solid foods and diet modifications throughout the infancy, this early colonization may shifts towards and adult-like microbiome, that may also be susceptible to changes by the use of medications such as antibiotics. Indeed, these drugs can alter the local ecosystem and disrupt the neonatal microbiome that could also be shaped by host genetics (4). Therefore, a variety of conditions can alter the human microbiota and its unbalance can potentially predispose the individuals to inflammatory diseases.

Usually, the intestinal microbiota plays an important role in a variety of physiological processes such as the maturation of host immunity, food fermentation and digestion, vitamin synthesis, regulation of intestinal hormones or host and drugs’ metabolism. These functions exerted by the gut microorganisms have in common the capacity to impact the inflammation and the immune response that are triggered in chronic diseases (5, 6). In fact, the microbial-mucosal immune system interactions are tightly regulated, mainly contributing to homeostasis and eubiosis conditions in the gastrointestinal tract (1, 2). This tolerogenic response in the gut is fundamental to avoid excessive reactions to the local commensal microbiota (or to food antigens) meanwhile maintains the immunological competence in a stand by condition, able to prompt react if a pathogenic invasion occurs. However, a combination of environmental factors such as a gastrointestinal infection and genetic susceptibility can lead to a tolerance breakdown and intestinal dysbiosis, with either decrease or expansion of certain commensals, reduced diversity with an increase in pathobionts, resulting in altered function of this ecosystem (7–9). The outcomes of these deregulated interactions at the intestinal mucosa can have systemic effects in host immunity and contribute to the development of the chronic inflammatory or autoimmune diseases, as outlined above (10, 11).

This Research Topic of Frontiers in Immunology, Mucosal Immunity section, included reviews, mini-reviews, hypothesis and theory, besides original research articles describing microbiome/metabolomics studies in mice and humans. These works evaluated the intestinal dysbiosis in inflammatory diseases as well as the crosstalk between the commensal microbes and the immune system. Furthermore, the role of the microbiota modulators (diet, pre-/probiotics, and fecal microbiota transplantation) have been discussed, focusing in the reestablishment of the gut immune homeostasis in chronic inflammatory conditions. A total of 24 articles accepted in this Research Topic involved contributions from 178 researchers and the main findings are briefly discussed in this editorial.

Considering the numerous molecules found in the gut ecosystem, quorum sensing represents small signaling molecules produced by bacteria, depending on the density of these microorganisms, with a subsequent regulation of their gene expression and bacterial physiology (12). These products are detected by microbes, interact with the host cells and contribute to gastrointestinal homeostasis (13). Coquant et al. showed the importance of the quorum sensing molecules, focusing on type I autoinducers, such as *N-*acyl-homoserine lactones (AHL). The authors pointed to the impact of these molecules on the innate and adaptive immune system, with important anti-inflammatory effects in experiments involving intestinal cell lines, thus indicating a perspective for the future use of natural or synthetic AHL to regulate gut inflammation and inflammatory bowel diseases (IBD).

Intestinal microbes may also produce short-chain fatty acids (SCFAs), which are end products from the fermentation of ingested dietary fibers. These molecules can bind specific receptors on mucosal cells and impact the local or systemic immunity, by inducing regulatory T cells (Tregs) and an anti-inflammatory response that control the exacerbated reactions in the gut mucosa, thus contributing to the maintenance of the barrier integrity (14, 15). Moreover, epithelial or mucosal immune cells express pattern recognition receptors (Toll-like receptors - TLRs, NOD-like receptors – NLRs, and others) that also contribute to the sensing of the host microbiota and regulation of inflammatory processes (16). Interestingly, on this Research Topic, Elias-Oliveira et al. emphasized the interactions between NLRs and gut microbiota, showing the dual role of these molecules in promoting inflammation or protecting against the harmful effects of dysbiosis-driven diseases, such as IBD, type 1 or type 2 diabetes and obesity.

Then, to emphasize the importance of gut microbiota in inflammatory conditions, three original research articles explored this subject in animal models (17–28). Watanabe et al. evaluated the importance of these microorganisms in chronic kidney disease (CKD), by treating C57BL/6 mice, which were submitted to unilateral ureteral obstruction (UUO), with broad-spectrum antibiotics. The depletion of gut microbes induced a decrease in IL-1β, IL-4, IL-6, GM-CSF, MIP-1β, fibronectin and type I collagen, improving the renal damage. Moreover, in Myd88^ΔIEC^ mice, the bacterial translocation to mesenteric lymph nodes, after UUO, was lower than in WT mice. The reduced expression of inflammatory cytokines and chemokines in Myd88^ΔIEC^ mice suggested that the role of the gut microbiota in CKD pathogenesis is related to the innate immune receptors’ signaling. The second study, performed by Omura et al. evaluated the gut microbiota in mouse models of acute flaccid myelitis and multiple sclerosis (MS), which are inflammatory conditions that affect the central nervous system (CNS). To achieve this goal, researchers infected SJL/J mice with Theiler’s murine encephalomyelitis virus (TMEV) and observed that although there were no significant differences in beta or alpha diversities between TMEV infected mice and controls, *Dorea, Marvinbryantia*, and *Coprococcus* genera changed after TMEV infection and correlated with immune molecules expression (T cell receptor, Immunoglobulin genes, CD109) in spinal cords. Lastly, Seo et al. demonstrated that the administration of an agonistic antibody to TREM-1 (triggering receptor expressed on myeloid cell-1) led to an improvement in dysbiosis, restored epithelial barrier and ameliorated colitis in mice. Besides that, the beneficial effect of α-TREM-1 was dependent on CD177+ neutrophils and macrophages, *via* TLR4/Myd88 signaling.

Dysbiosis observed in autoimmunity is associated with impaired epithelial barrier function, systemic inflammation and decreased regulation in the gut mucosa (29–32), as a result of a variety of mechanisms stimulated or amplified by a dysbiotic microbiota (31, 33). In a review article from Brown et al. authors compiled data on intestinal dysbiosis in systemic lupus erythematosus, rheumatoid arthritis, and multiple sclerosis, discussing the contribution of the gut microbiota and SCFAs, with an additional special focus on recent studies linking the molecules from tryptophan metabolism to the protection or induction of autoimmune diseases. In addition, Hou et al. revised the intricate communication among leukocytes, intestinal stem cells (ISC) and gut microbiota, showing that this interaction is extremely important for the regenerative capacity of the gut epithelial cells. Indeed, signals derived from gut microbes affect the ISC development; i.e., while Tregs induce ISC renewal, the Th1/Th2/Th17 lymphocytes inhibit ISC activity. Besides that, authors discussed the importance of organoids derived from ISC to study this crucial interaction that could provide promising treatments for IBD.

Still on IBD, epidemiological studies showed higher incidence of IBD in patients with chronic obstructive pulmonary disease (COPD), and intestinal dysbiosis in patients with respiratory diseases, pointing to the importance of the gut-lung axis in health and disease (34–36). Two reviews explored this connection in the context of IBD *vs.* COPD and in COVID-19. Raftery et al. reviewed what is considered a “healthy microbiota” in the human gut and lungs. They also showed that intestinal dysbiosis (*via* bacteria metabolites) affects the lung homeostasis and may result in systemic effects on the immune system or production of inflammatory cytokines. De Oliveira et al. reviewed the gut-lung axis concepts and suggested that intestinal dysbiosis may affect the immune responses in the lungs and disease severity in COVID-19. Preliminary studies have shown the persistence of SARS-CoV-2 in the gastrointestinal system, and not only intestinal dysbiosis, but also nasopharyngeal and pulmonary dysbiosis in COVID-19 patients.

In a human cross-sectional study, Cortez et al. detected differences in the gut microbial composition between pediatric patients with primary sclerosing cholangitis (PSC) and those with PSC along with ulcerative colitis (UC). In patients under 10 years old, *Streptococcus* and *Veillonella* genera were increased in the PSC group when compared with controls, while *Veillonella* and *Escherichia-Shigella* were higher in the PSC+UC subjects. The increased *Veillonella* was associated with active disease, and this genus, in addition to *Megasphaera*, correlated with bilirubin and gamma-glutamyl transferase. These results suggested an association between disease activity and intestinal microbiome, thus opening new avenues for the diagnosis and prognosis of pediatric PSC. In another study, Xu et al. detected dysbiosis and increased intestinal permeability in patients with encephalitis, with predominance of the Proteobacteria phylum, and *Parabacteroides* and *Oscillospira* genera, besides increased plasma concentrations of D-lactate, intestinal fatty acid-binding protein, lipopolysaccharides, and lipopolysaccharide-binding protein. Furthermore, the levels of SCFAs (acetate, propionate and butyrate) were decreased in fecal samples from patients, while there was a direct correlation between the cerebrospinal fluid/serum albumin ratio and Ruminococcaceae relative abundance. Altogether, these data indicated that gut microbiota and their metabolites could play a role in brain injury and encephalitis’ severity.

Regarding the applicability of microbiota modulators in inflammatory conditions, several original research articles investigated this question in experimental models. In autoimmune diseases, Kim et al. analyzed the effect of multi-strain probiotics (IRT5) containing *Lactobacillus acidophilus, L. casei, L. reuteri, Bifidobacterium bifidum*, and *Streptococcus thermophiles* in non-obese diabetic (NOD) mice. Compared to controls, the IRT5 supplementation during 36 weeks decreased the diabetes incidence, pancreatic inflammation, insulin autoantibodies levels, intestinal barrier permeability, and increased the β-cell mass in NOD mice. Gusmao-Silva et al. administered probiotic bacteria (*Lactococcus lactis* NCDO 2118) carrying a recombinant protein (Hsp65) in chronic and acute mouse models of arthritis, and showed that this combination suppressed arthritis onset in both models and induced regulatory CD4^+^LAP^+^ T cells. In addition, the probiotic supplementation decreased IL-17, IFN-γ, anti-type II collagen autoantibodies, rheumatoid factor, and promoted an increase in beneficial microbes in the gut, including *Bifidobacterium* and *Lactobacillus*. The main findings of Sato et al. study were the beneficial effects of the *Lactobacillus jensenii* TL2937 in the experimental colitis induced by dextran sodium sulfate. Upon treatment with this *Lactobacillus* strain, mice mice presented reduced disease and myeloperoxidase activity, decreased pro-inflammatory cytokines, and increased regulatory IL-10 and IL-27 production in the intestine.

In allergic diseases, Yazdi et al. examined the impact of probiotic (*Lactobacillus rhamnosus* GG) administration alone or in combination with a prebiotic (turmeric powder) in asthma induced by house dust mite in BALB/c mice. The synbiotic administration decreased the number of eosinophils in bronchoalveolar lavage fluid, the Th2 lymphocytes, IL-5 and IL-13 levels, serum IgE concentrations, and hyper responsiveness to methacholine.

Lastly, to investigate the effect of obesity and intestinal dysbiosis in sepsis, Panpetch et al. orally administered (1→3)-β-D-glucan (*Candida albicans*) in mice fed with high-fat diet, and then induced sepsis by cecal ligation and puncture. In the obese mice, which previously received β-D-glucan from *Candida albicans*, sepsis was more severe, with liver and kidney damage, endotoxemia, increased intestinal permeability, inflammatory cytokines, and gram-negative bacteria in the gut. Researchers also evaluated the effect of *Lactobacillus rhamnosus* L34 probiotic on sepsis, and observed an improvement in disease severity, with a decrease in the intestinal permeability, and an increase in anti-inflammatory butyrate-producing commensals in the gut.

In addition, four reviews explored the mechanisms and the impact of probiotics in the modulation of the immune responses in human inflammatory conditions. A review by Yan and Polk focused on the impact of probiotics and postbiotics in intestinal homeostasis and epithelial barrier function, mucosal immune responses, and the gut-brain axis communications. Moreover, authors discussed the beneficial effects of probiotics or their metabolites in IBD and colorectal cancer, thus proposing personalized treatments based on the individual microbiome profiles. In another important study, Cristofori et al. explored the complex interactions among diet, microbiota, their metabolites and the immune system, reviewing key concepts about microbial-human evolution, hygiene hypothesis, microbiota resilience and dysbiosis. Furthermore, the probiotics’ immune regulatory mechanisms in the intestine were discussed, with examples of their application in several diseases, including IBD, irritable bowel syndrome, celiac disease, obesity, autism, and Parkinson’s disease, evidencing why the intestine can be considered “a door to the body”. Marietta et al. compiled a complete and up-to-date information on the clinical trials based on bacterial monotherapies or multistrain probiotic treatments, as well as fecal microbiota transplantation (FMT) in several autoimmune diseases, offering a perspective on the implications and translation of experimental studies from animals to humans. The highlight of the review is the discussion on the possible use of FMT in the treatment of autoimmune diseases, including celiac disease, multiple sclerosis, rheumatoid arthritis, type 1 diabetes and Sjögren’s syndrome. Lastly, Pecora et al. reviewed the role played by intestinal microbiota in celiac disease pathogenesis and the effects of probiotic supplementation in improving symptoms of these patients, drawing attention to the importance of carefully evaluating which probiotic to use, the dose and the length of treatment. In addition to these excellent reviews, De Oliveira et al. showed the beneficial effect of probiotics in respiratory infections, and discussed their possible use as an adjunctive therapy in COVID-19.

In a very interesting hypothesis-driven article, Ouyang et al. revisited the beneficial properties of *Akkermansia muciniphila* in supporting an anti-inflammatory milieu in the gut, preventing increased intestinal permeability, bacterial translocation and systemic inflammation. Most interestingly, the authors discussed the application of *A. muciniphila* supplementation in metabolic diseases and cancer, as well as alternatives to increase the abundance of this synbiotic microbe in our guts. Finally, researchers hypothesized that supplementation with *A. muciniphila* in patients living with HIV could decrease the barrier permeability, bacterial translocation, inflammation, and the risk to develop cardiovascular diseases or type 2 diabetes.

Lastly, the influence of diet on shaping the gut microbiota was also addressed in three studies; two reviews and one original research article. Basson et al. focused on the role of dietary fatty acids and high-fat diets in modulating the immune system *via* alterations of the gut microbiota and epithelial barrier permeability, reviewing mainly IBD studies. Kahalehili et al. evaluated the role of indole-3-carbinol (I3C), phytochemicals found in cruciferous vegetables, in modulating autoimmune diabetes through aryl hydrocarbon receptor (AhR) ligands. The metabolized I3C from the diet induced AhR activation in the small intestine, alterations in microbiota diversity, differentiation of Th17 cells in the lamina propria, along with insulitis in non-obese diabetic (NOD) mice. In a very interesting transkingdom network analysis, to predict host-microbiota interactions, several Firmicutes phyla members were negatively regulated by I3C. Furthermore, by utilizing AhR knockout mice, they observed that *Intestinimonas* is downregulated by AhR activation. In addition to these works, Raftery et al. discussed the role of microbial-metabolized dietary metabolites, showing that the intake of saturated fats induces an increased intestinal permeability and inflammatory response. On the other hand, high soluble fiber diet, rich in vegetables and fruits, is able to suppress inflammatory mediators *via* SCFAs (mainly butyrate), thus inducing regulatory mechanisms and protective effects in IBD or COPD.

Collectively, the articles from this Research Topic showed important aspects of the role played by intestinal dysbiosis in inflammatory diseases. Furthermore, the strategic approaches for the use of commensals or microbiota modulators, and consequently their involvement in immune regulation were discussed and contributed to increase the knowledge in the field.

## Author Contributions

GO wrote the initial draft of the editorial. AF, CC, and VT revised and approved the manuscript. All authors contributed to the article and approved the submitted version.

## Conflict of Interest

AF holds stocks in Alba Therapeutics and receives financial support from Takeda Pharmaceuticals. VT has received financial support from Elysium Health and Evelo Biosciences.

The remaining authors declare that the research was conducted in the absence of any commercial or financial relationships that could be construed as a potential conflict of interest.

## Publisher’s Note

All claims expressed in this article are solely those of the authors and do not necessarily represent those of their affiliated organizations, or those of the publisher, the editors and the reviewers. Any product that may be evaluated in this article, or claim that may be made by its manufacturer, is not guaranteed or endorsed by the publisher.
